# Differential Gene Expression and Biological Analyses of Primary Hepatocytes Following D-Chiro-Inositol Supplement

**DOI:** 10.3389/fendo.2021.700049

**Published:** 2021-07-15

**Authors:** Feier Cheng, Shao-jun Yun, Jin-ling Cao, Ming-chang Chang, Jun-long Meng, Jing-yu Liu, Yan-fen Cheng, Cui-ping Feng

**Affiliations:** ^1^ College of Food Science and Engineering, Shanxi Agricultural University, Taigu, China; ^2^ Shanxi Research Station for Engineering Technology of Edible Fungi, Shanxi Agricultural University, Taigu, China

**Keywords:** primary hepatocyte, D-chiro-inositol, RNA‐Seq, insulin resistance, molecule mechanism

## Abstract

Dietary supplements have improved the prevention of insulin resistance and metabolic diseases, which became a research hotspot in food science and nutrition. Obesity and insulin resistance, caused by a high-fat diet, eventually result in severe metabolic diseases, can be prevented with the dietary supplement D-chiro-inositol (DCI). In this work, we isolated mice primary hepatocytes with palmitic acid stimulation and DCI was applied to compare and contrast its effects of in primary hepatocyte biology. Before and after intervention with DCI, we used RNA-Seq technology to establish a primary hepatocyte transcriptome gene profile. We found that both PA and DCI cause a wide variation in gene expression. Particularly, we found that DCI plays critical role in this model by acting on glycolysis and gluconeogenesis. Overall, we generated extensive transcripts from primary hepatocytes and uncovered new functions and gene targets for DCI.

## Introduction

Adjustments in diet structure play a crucial role in improving human health and disease. Excessive energy intake can easily induce obesity, which causes insulin resistance, that is becoming a serious problem that threatens human health, due to increased type 2 diabetes incidence of and other metabolic diseases (1–3). Therefore, it is necessary to adjust the diet composition and control calories to improve insulin sensitivity and prevent diseases that are caused by increased caloric intake, such as diabetes and cardiovascular disease (4, 5). At present, we have not been able to grasp the potential molecular processes that drive the physiological and metabolic differences caused by high-fat diet intake.

The metabolic regulator of the human body is the liver and dietary intake is the main link in the body’s energy supply (6, 7). The liver needs to metabolize several substances to make effective use. Therefore, changes in diet can cause changes in the metabolic mechanism of the liver. The liver energy balance and glucose homeostasis are primarily maintained by the regulation of insulin concentration. Excessive fat intake can lead to obesity and insulin resistance (8), which can affect liver blood sugar regulation, leading to hyperglycemia (9). The adjustment of diet structure and intake can reduce the abnormal accumulation of fat in the liver, improve glycogen regulation and reduce inflammation (10, 11). Therefore, analyzing the effects of dietary changes on liver regulation can improve our understanding of the liver metabolic regulation.

D-chiro inositol (DCI) is an epimer of inositol that is abundant in plants (12, 13), such as soybean (14), buckwheat (15) and pumpkin (16). At present, the method of batch preparation of DCI is through natural product extraction and the main test material is *Fagopyrum esculentum Moench*. It is a combination of edible and fake plant resources (17), in comparison with common cereal crops, such as wheat, rice and oats that are rich in D-chiro-inositol. In the human body, endogenous DCI is mainly derived from dietary intake (18) and inositol is converted into its stereoisomer by a putative epimerase. Buckwheat has received increasing attention due to its therapeutic effects on metabolic diseases (19), such as diabetes (20) and hypertension (21). It has been suggested that DCI improves endothelial dysfunction and has antioxidant activity in diabetic rats (22). DCI is mainly distributed in the blood and tissues sensitive to peripheral targets, such as the liver and fat. Studies have shown that elevated levels of DCI in plasma, liver, muscle and fat can improve PCOS or diabetes (23, 24). These results suggest that insulin resistance is susceptible to tissue DCI deficiency; therefore, DCI supplementation may have an effect on insulin sensitivity (25, 26). Previous laboratory studies have shown that DCI can enhance insulin sensitivity and regulate energy metabolism (27, 28).It was also found that DCI exhibits pyruvate dehydrogenase activity by inactivating the pyruvate dehydrogenase kinase (PDK) (29), which increased the possibility that DCI could transfer mitochondrial pyruvate to oxidation in liver cells; thereby, limiting the gluconeogenesis of pyruvate carboxylation (30).

With the rise and application of RNA-Seq technology, a relatively comprehensive and in-depth sequencing technology, that analyzes the transcriptome, was born (31–33). This application has given a new understanding and direction in understanding the complexity and specificity of substances. To some extent, RNA-Seq technology compared with other methods is more efficient, accurate and provides detailed analysis of the results. The transcriptome is called the “group” largely due to its own integrity that allows RNA analysis of cells at different growth periods and conditions, rendering personalized customization possible for a variety of tests (34, 35). Analyzing the transcriptome is essential for interpreting the functional components of the genome and in revealing the molecular components of cells and tissues, and for understanding development and disease. In order to fully reveal the mechanism of D-chiro-inositol’s high efficiency in stabilizing blood glucose in animal experiments, we established the pa-induced mRNA gene profile of mouse primary hepatocytes by RNA-Seq technology and explored the detailed mechanism of natural compounds that are involved in regulating the expression levels of key genes and proteins. The liver is the main organ of biotransformation and protein synthesis, where small molecules and natural active substances play a main role. The liver was studied using mice that are susceptible to the body’s regulating factors of intervention, such as D-chiro-inositol. In this study, we investigated the effect of D-chiro-inositol on the cause of insulin resistance, induced by PA, in primary hepatocyte cell model to determine the interaction between the two molecules and other biological processes.

## Method

### Primary Hepatocytes

The primary hepatocytes were isolated with HBSS (Hyclone, Logan, UT, USA) and collagenase IV (Gibco, Grand Island, NY, USA) (36, 37). The plates used were pre-coated with rat tail collagen I (BD Biosciences, China), and the cells seeded in DMEM LOW (Hyclone, Logan, UT, USA), supplemented with 10% Fetal bovine serum (FBS)(Gibco, Grand Island, NY, USA), 1% 2-hydroxyethyl, 0.5 μg/ml hydrocortisone, 0.5 μg/ml insulin (Gibco, Grand Island, NY, USA) and 100 units/mL of penicillin/streptomycin. After attachment (4 h), the medium was removed, and the hepatocytes incubated (20 h) in medium the above without FBS. We incubated the hepatocytes for 24 h with PA (100 μM, Sigma) to generate an insulin resistance model and for early intervention with DCI (10 μM) (purity > 98%, Yuanye BioTechnology Co., Shanghai, China) which was dissolved in PBS.

### RNA Extraction

Total RNA was extracted from primary hepatocytes using Trizol (Trizol, Takata, Japan) and according to the manufacturer instructions. Three PA and three DCI samples were prepared. The samples were quantified by Nanodrop 2000 spectrophotometer (Thermo Fisher Scientific) and the RNA qualitatively controlled by agarose gel electrophoresis and the Agilent 2100 biological analyzer (Agilent Technologies Inc. CA, USA). All samples showed concentrations greater than 150 ng/μL and total RNA (RIN) levels were greater than 7 (showed in supplement).

### RNA Seq

The establishment of the database was carried out at Genedenovo Biotechnology Co., Ltd (Guangzhou, China). The RNA-seq library was performed using NEBNext^®^ Ultra ™RNA Library Prep Kit for Illumina^®^ (NEB#7530, New England Biolabs) and the 150 bp paired terminal sequencing was performed on the Illumina HiSeq 4000 platform. The sampling read quality and potential contamination were performed using FastQC v0.11.5 detection and filtration fittings. Next, the clean reads were aligned to the *Mus musculus* genome (GRCm38.p6) with fast and sensitive Tophat2 (v2.1.1) (37), using *Mus musculus* genome index and including transcript structures. The Differential expression analysis was performed by edgeR (v3.12.1). A quantitative method for gene expression was a transcribed fragment read per million bases per million markers (FPKM).

### Screening of Differentially Expressed Genes (DEGs)

We used pheatmap package in R software for heatmap analysis. Genes with fold change ≥ 2 and false discovery rate (FDR) of < 0.05 were considered significant DEGs. After that, the DEGs were subjected to enrichment analysis of the GO function and the KEGG pathway. GO and KEGG pathways with a Q-value ≤ 0.05- corrected *P*-value were significantly enriched in DEGs.

### Quantitative Real Time PCR

To generate cDNA, the extracted RNA was subjected to RT-PCR, according to the SYBR^®^ Premix Ex Taq II (TAKARA BIO INC. Japan) reagent instructions and using an ABI 7500 Real-Time PCR machine (Life, Qiagen, Germany). The primer sets used for the quantitative real time PCR analysis are shown in **Table 1**.

**Table 1:** Quantitative real-time PCR primers list.

Primer name	Sequence	Size	Accession
Hkdc1	F: ACAAGCTGCACCCGCACTTCTC	158	NM_145419.1
	R: GTCCTTCCGTGGCTGCTGTAAC		
Pik3cg	F: GAGAGTGCCCTTCGTCCTAAC	126	NM_001146200.2
	R: TGATGGCGAAGAGCTAGGTAAG		
Slc2a2 (Glut2)	F: AGATTGGGCCAGGTCCAATCC	132	NM_031197.2
	R: ACTGGAAGCAGAGGGCGATGAC		
Pik3r3	F: AAATGGAGAGGCCAAGCAGTAG	137	NM_001355584.1
	R: GTGCCTGACAACAAACCCAAAC		
Socs1	F: CTTCCGCTCCCACTCCGATTAC	173	NM_001271603.1
	R: GCGCGAAGAAGCAGTTCCGTTG		
Abcc8	F: TTCGAAGGGCGCATCATCATTG	120	NM_001357538.1
	R: GAATCTGATGGTGCCGCTGAAG		
Pklr	F: GAGGCTGTCTGGGCAGATGATG	114	NM_001099779
	R: TCGCCAGCCTGTCACCACAATC		
Pgf	F: GCCTAGAACCTGCCCTGATTCC	120	NM_001271705.1
	R: GCCGAATGTCCTGTCCCATCTC		
Fgfr4	F: TTGACCTCCGCCTGACCTTTG	142	NM_008011.2
	R: GTCGTCTGCGAGTCAGAGAAAG		
Csf1	F: GGGAATGTGGCCTACCACTAC	162	NM_001113529.1
	R: CTCCAGGGCCCACAATAAATAG		
Lama4	F: CCATCACCTCCGCTTCTCAAAC	153	NM_010681.4
	R: GGCGGACTTCAAAGGCAATTTC		
Lama3	F: TCACGTGGTCCTAGCCAATTC	145	NM_001347461.1
	R: CTTCCCTGCCTCCATGTAGAC		
Itgb8	F: GATGACTTCTCCTGTCCCTATC	116	NM_177290.3
	R: GATGGGCACTGACACCGATCTC		
Itga4	F: GGAAGCCAGCGTTCATATTCAG	155	NM_010576.4
	R: AAACATGGGCCACGTTCTCATC		
Creb3l1	F: TGTGCCCTGCCTTCCTGCATTC	103	NM_011957.2
	R: GCTTCGGGAAGGCATCTGACTG		
Efna3	F: GCATCGCCTTCTTCCTCATGAC	135	NM_010108.1
	R: CGCTCCCAACCATGGGAAGAAG		
Csf3r	F: CTGGAGCCCGCCAGTTTGTATC	175	NM_001252651.1
	R: TGCAGCAGAGCCAGGTCACTAC		
Gys2	F: TGTGGCTGACCCTACTGCATAC	123	NM_145572.2
	R: CTTTGCCGGCGTGACTGTTTAC		
Fgf21	F: GGTGTCAAAGCCTCTAGGTTTC	139	NM_020013.4
	R: CATGGGCTTCAGACTGGTACAC		
Il2rg	F: TGCACTGGAAGCTGTGCTTATC	186	NM_001308535.1
	R: AGACTCTCAGTCAGCCCTTTAG		
Angpt2	F: AGAGTACTGGCTGGGCAATGAG	109	NM_007426.4
	R: ATACAGCGAATGCGCCTCGTTG		
Comp	F: AGCCTTCAACGGCGTGGACTTC	107	NM_016685.2
	R: CGTAGAAACTGGAGCTGTCTTG		
Pdgfd	F: GGCAACTGTGGTTGCGGAACTG	174	NM_001357397.1
	R: GTCACATCGCTCATGATGATCC		
Lpar1	F: CTCCTGGCCGAGTTCAACTCTG	108	NM_001290486.1
	R: GTTCTCGTTGCGCTGGCAACAC		
G6pc (G6Pase)	F: ATGCCAGCCTCCGGAAGTATTG	136	NM_008061.4
	R: GGCCGCTCACACCATCTCTTAG		

### Immunofluorescence

The inoculated cells were washed 2-3 times with PBS. Two mL of 4% Paraformaldehyde was added to each well of a 6-well plate and fixed at 4°C for 30 min. The solution was changed or directly added to PBS for shaking 2 to 3 times. Subsequently, the blocking and incubation of the antibody were carried out according to the standard of specification and the dilution concentration of the selected antibody in the pre-experiment and incubated for 1.5 to 2 h. After recovering the primary antibody, the mixture was shaken and washed 2 to 3 times, and the fluorescent secondary antibody was incubated in the dark. DAPI was used to stain the nuclei and the excess dye was discarded. After washing with PBS, the tablets were sealed and developed.

### Western Blot

An equal amount of the sample was subjected to SDS-PAGE electrophoresis and then the protein was transferred to a 0.45 μm-well PVDF membrane (Millipore Co., Ltd.) (90 V, 90 minutes). The skimmed milk powder was sealed for 2 hours. The primary antibody required was incubated overnight at 4°C and the corresponding secondary antibody for 1.5-2 hours at room temperature on a shaker. After washing with TBST 2-3 times, an appropriate amount of ECL luminescent reagent was uniformly added to the PVDF membrane and developed in a Chemi doc + XRS system. The gray scale analysis was performed using the image Lab software to calculate the optical density ratio of the target.

### Statistics

All data presented in the results were analyzed by Graphpad Prism 6, ANOVA for univariate analysis, Tukey’s multiple comparison and the results were analyzed from 3 or more independent experiments using mean ± standard error (AVG ± SEM). In the statistics, *p* < 0.05 was significantly different and *p* < 0.01 was extremely significant.

## Results and Discussion

### RNA Seq and Read Mapping

**Figure 1:**
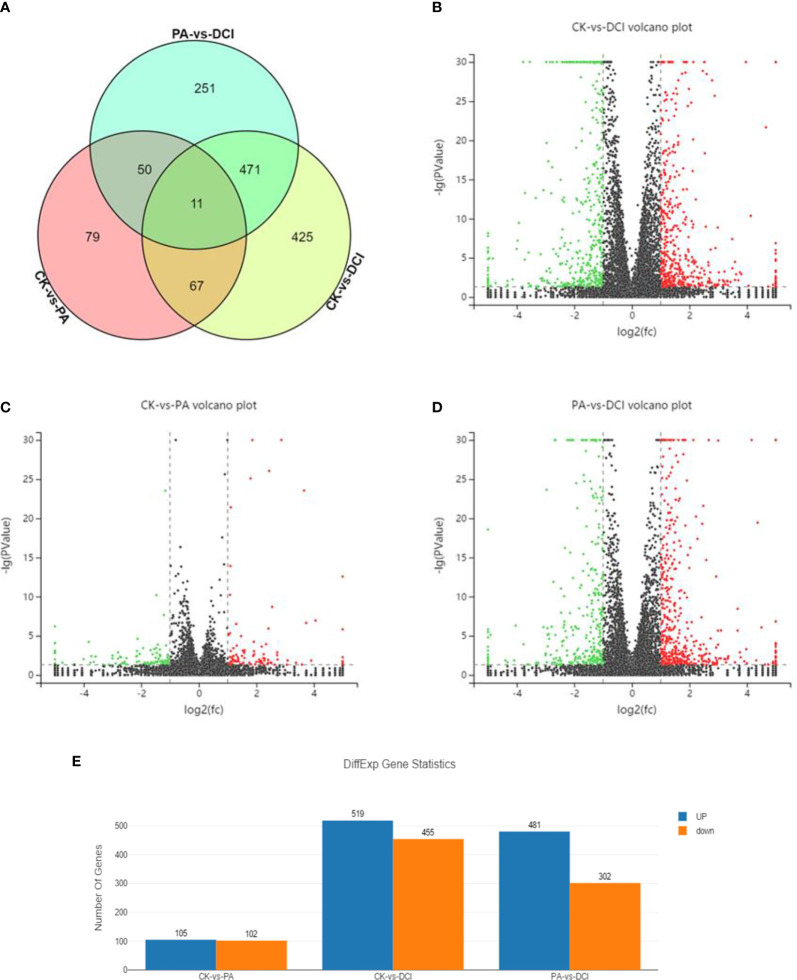
Venn diagram and volcano plot of DEGs between different groups using RNA sequencing. Venn diagram shows the distribution of DEGs among three groups. CK, control group; PA, PA-treated group; CK, PA, and 10 μM D-chiro-inositol cotreated group. Red, green, and black indicate the downregulation, no significant change, and up-regulation, respectively. **(A)** Venn diagram; **(B–D)** Volcano plot of differentially expressed genes between different groups; **(E)** The number of differentially expressed genes.

RNA seq analysis was performed to determine the expression profile of mRNA in primary hepatocytes upon DCI supplementation. A total of 37.92 and 54.87 million reads were aligned to the *Mus musculus* reference genome (GRCm38.p6) in the PA and DCI groups, respectively. A total of 1354 differentially expressed genes (DEGs) were obtained from the 3 treatment groups (**Figures 1A–D**). The comparative study between different treatment groups found that in the comparison of differential genes between CK and PA group, 105 genes were up-regulated, and 102 genes were down-regulated (**Figure 1E**). Compared with the DCI group, the number of significantly up-regulated genes was 481 and the number of down-regulated genes was 302.The above results indicated, that compared with CK group, the PA treatment of mice primary hepatocytes could change the expression of many genes, and that DCI intervention could regulate the gene transcription.

**Figure 2:**
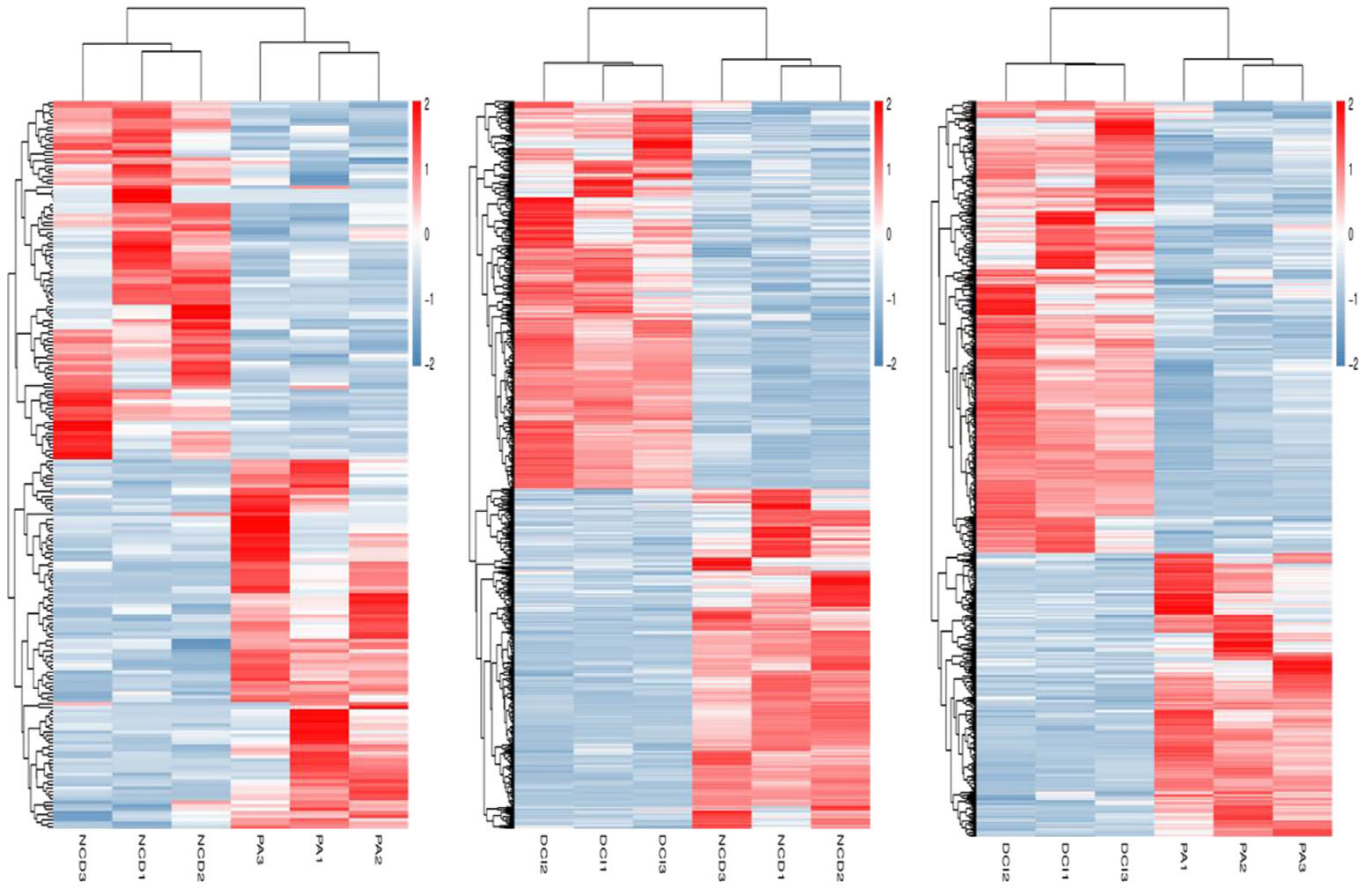
Heatmap of differentially expressed genes profiling between three different conditions *via* RNA-Seq.

The relevant heatmaps of DEGs in the sequencing results were analyzed to establish heatmaps of DEGs in primary hepatocytes under different treatment methods in the CK group, PA group and DCI group (**Figure 2**).

### Pathway Enrichment Analysis in DCI Induced DEG

**Figure 3:**
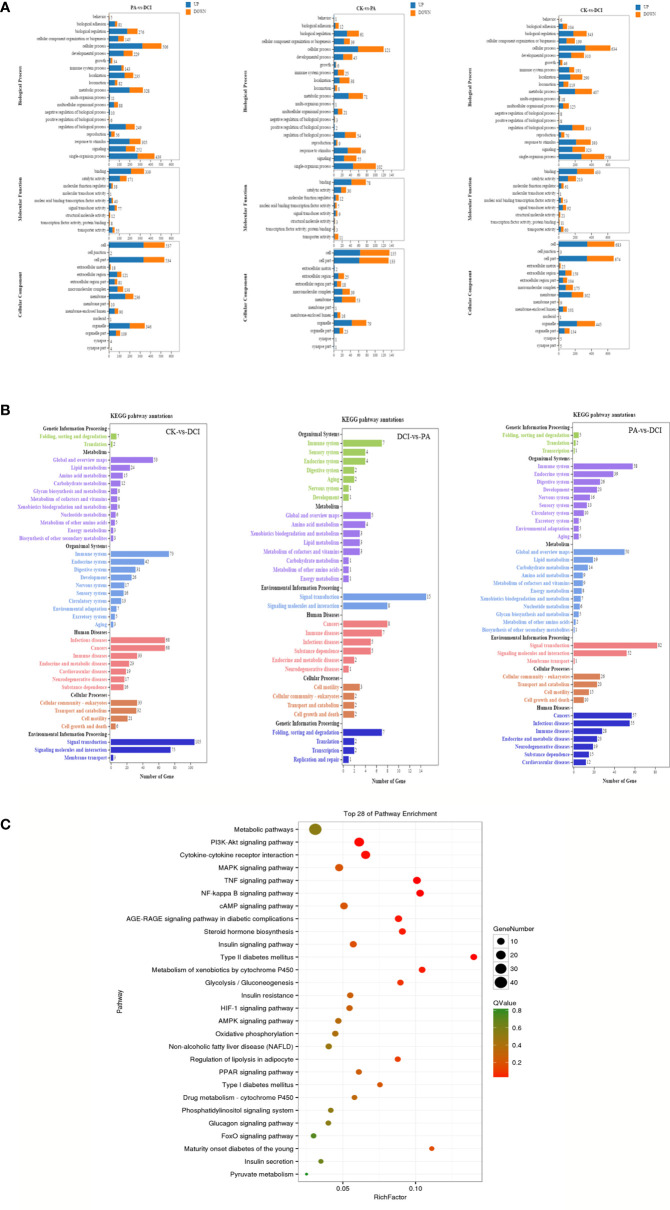
GO/KEGG Pathways of the differentially expressed genes. **(A)** GO terms for the DEGs; **(B)** KEGG terms for the DEGs; **(C)** The top 28 ranging KEGG pathways of the DEGs. Canonical signaling pathway (KEGG) enriched under DCI induction. control group(CK), PA treated group (PA), and 10 μM DCI and PA cotreated group (DCI).

At the same time, the DEGs of different treatment groups were classified. In the GO library, we can compare the research data with the existing mouse sequencing data in the library and classify the differential in genes expression into annotations that are based on the molecular function (MF), biological processes (Biological Process, BP) and cellular components (CC). By comparison (**Figure 3A**), D-chiro inositol gene regulation in primary liver cells was mainly concentrated on biological processes, including biological regulation and cellular, metabolic and single organism processes. Based on the mRNA-gene regulatory information, KEGG was used to analyze a significantly enriched mRNA to clearly study the biological effects of gene differences between different groups (**Figure 3B**). The study found that the major enriched signaling pathways were Lipid metabolism, energy metabolism, pyruvate metabolism, PI3K-Akt, MAPK, TNF and glucose signaling pathways. The number of differential genes enriched by cAMP, FoxO signaling pathway and Glycolysis/Gluconeogenesis was higher (*p* < 0.05). This is consistent with our previous studies that found that D-chiro inositol is an insulin sensitizer, further confirming the reliability of sequencing results (**Figure 3C**).

### Effect of D-Chiro Inositol on Translocation of PKC ϵ Protein Membrane

**Figure 4:**
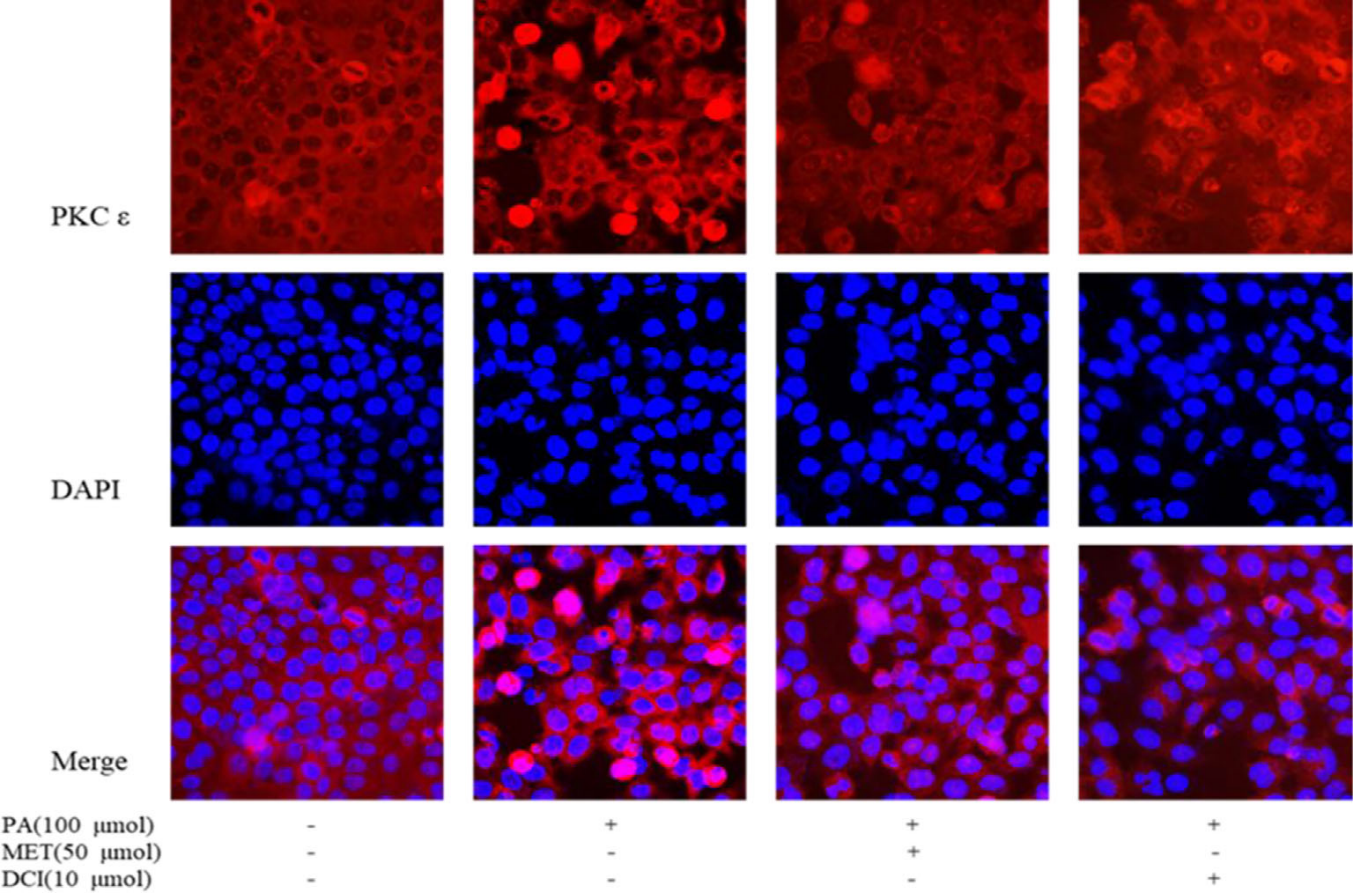
The effect of D-chiro-inositol on the expression of PKC in the PA-induced primary hepatocyte ( ± SD, n≥3).

As shown in **Figure 4**, the changes of intracellular proteins were investigated by immunofluorescence staining. The expression of PKCϵ is shown by red fluorescence and the nucleus that was stained by DAPI in blue. The results showed that the expression of PKCϵ on the cell membrane was significantly increased under PA stimulation. The intervention of MET and DCI inhibited the expression of PKCϵ on the cell membrane.

### Effect of D-chiro Inositol on the Expression of Related Proteins in PI3K/AKT Signaling Pathway

**Figure 5:**
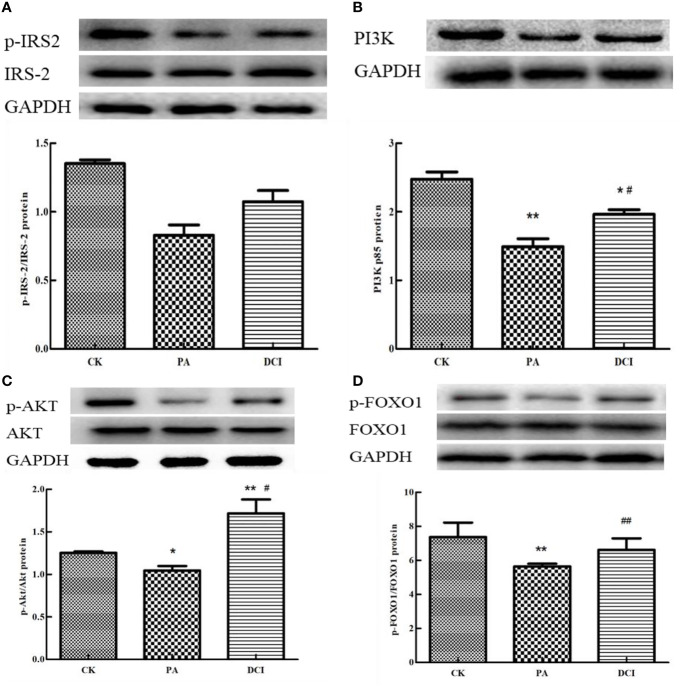
The effect of D-chiro-inositol on insulin resistance signal pathway in the PA-induced primary hepatocyte ( ± SD, n≥3). Representative western blots expression and the protein bands were quantified by densitometry of **(A)** p-IRS-2/IRS-2; **(B)** PI3K; **(C)** p-AKT/AKT; **(D)** p-FOXO1/FOXO. *p < 0.05, **p < 0.01 compared with the CK group; ^#^p < 0.05, ^##^p < 0.01 compared with the PA group.

The main proteins in the upstream and downstream of PI3K/AKT signaling pathway were selected and their protein expression levels determined. The protein sites related to IRS-2, PI3K, AKT and FOXO1 were involved in the establishment of insulin resistance model and we aimed at determining the effective regulatory role of DCI in this process. As shown in **Figure 5**, PA treatment of mouse primary liver cells significantly reduced p-IRS-2, AKT and FOXO1. Compared with the PA-induced group, the DCI treatment group significantly up-regulated p-IRS-2, AKT and FOXO1. At the same time, DCI significantly up-regulated the expression of PI3K protein (*p* < 0.05).

### Effect of D-chiro Inositol on the Expression of Genes Related to Insulin Resistance Pathway

**Figure 6:**
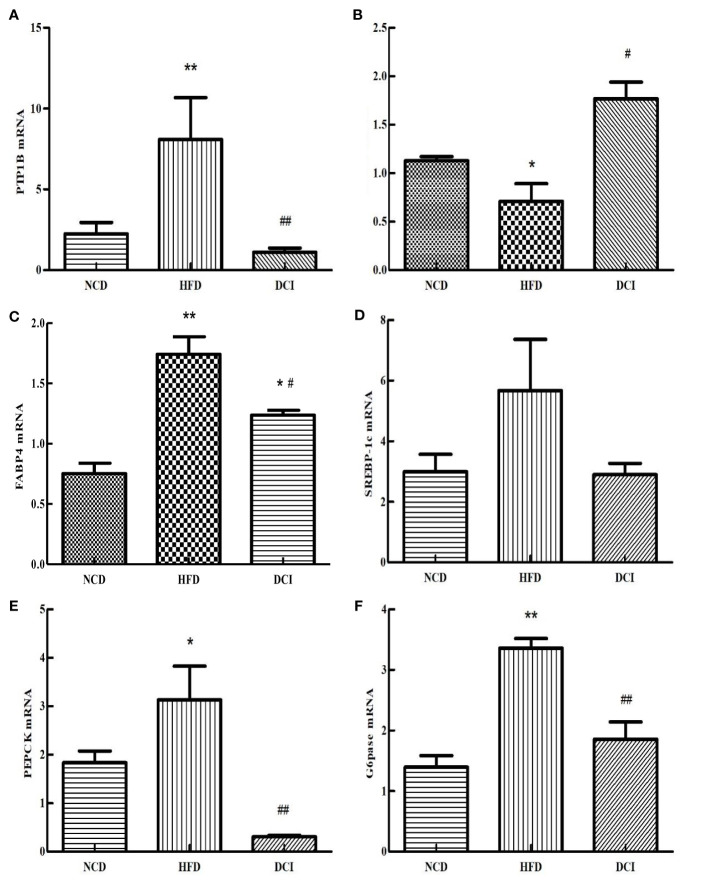
The effect of D-chiro-inositol on insulin resistance signal pathway gene in the PA-induced primary hepatocyte ( ± SD, n≥3). **(A)** *PTP1B* mRNA expression; **(B)** *XBP1S* mRNA expression; **(C)** *SREBP-1c* mRNA expression; **(D)** *FABP4* mRNA expression; **(E)** *PEPCK* mRNA expression; **(F)** *G6pase* mRNA expression. *p < 0.05, **p < 0.01 compared with the CK group; ^#^p < 0.05, ^##^p < 0.01 compared with the PA group.

According to the RT-PCR results (**Figure 6**), the expression of *PTP1B* was significantly increased in the PA group compared with the CK group (*p* < 0.05). As for the PA group, the expression of *PTP1B* was significantly decreased in the DCI group (*p* < 0.05). At present, many studies are shifting the focus of *XBP1S* research to the regulation of glucose homeostasis. *SREBP-1c* acts as an important site in insulin signaling. The *FABP4* can improve the sensitivity of insulin and is a possible target for T2MD. As shown in **Figures 6C, D**, the gene expression of *SREBP-1c* and *FABP4* in the PA group was significantly increased compared to the CK group (*p* < 0.05); while, the gene expression in the DCI group was significantly decreased compared to the PA group (*p* < 0.05). *G6Pase* is a key rate-limiting enzyme that regulates hepatic gluconeogenesis. *Pepck* also plays a role in the regulation of gluconeogenesis, effectively utilizing non-sugar substances in the process of raising blood sugar level and achieving its active transformation and energy supply. As shown in **Figures 6E, F**, the gene expression of *PEPCK* and *G6pase* in the PA group was significantly increased compared to the CK group (*p* < 0.05); while, the expression of the gluconeogenesis pathway negative regulator gene in was significantly higher in the DCI group. (*p* < 0.05).

Although DCI can play a leading role in the liver metabolic regulation and blood glucose stability, no studies have reported the effect of DCI on gene expression profiles of primary hepatocytes. Our findings indicated that DCI helps in keeping the insulin resistance in relation to signaling pathways. The DCI metabolism is associated with insulin sensitivity, and this view supports the notion that DCI acts as a second messenger of insulin response. In addition, DCI supply has been previously shown to reduces liver weight and our data is consistent with the observations by Larner, J (38).

The previous studies showed that PA-stimulated cells trigger oxidative-stress and related metabolic disorders. For example, PA stimulates HepG2 cells resulting in glucose metabolic disorders (25). In our experiments, it was found that 100 μM PA stimulated mouse liver primary cells to trigger hepatic glucose output disorder, indicating that high concentrations of PA can induce insulin resistance in primary cells. In addition, DCI has been found to prevent PA-mediated endothelial dysfunction (28). At the same time, it has been reported that DCI can activate pyruvate dehydrogenase to exert a role in improving glucose disorder and lipid metabolism in mice. In our study, DCI was shown to restore PA-induced signaling pathways involved in in primary liver cells’ insulin resistance.

In this study, 1354 DEGs were obtained. As a natural sugar alcohol compound, DCI can protect against glucose-lipid metabolism disorder, induced by PA in mouse liver primary cells. By using high-throughput RNA seq technology, the gene expression changes in primary hepatocytes, under the intervention of DCI, were explored. Based on the results, 1354 unique DEGs were detected in the DCI supplementation, many of which are involved in biological regulation and metabolic processes (e.g. cell regulatory processes and biological regulation) and are primarily located in the cell membrane and cytoplasm. In addition, KEGG analysis revealed several metabolically related pathways (PI3K/AKT, MAPK, glucose metabolism pathway), FoxO and immune response-related signaling pathways. The results of the enrichment analysis also provided assurance of the accuracy and a certain degree of support for the study results. The results of pathway enrichment showed that the up-regulated DEGs were mainly enriched in the PI3K/AKT pathway. In 1985, Phosphoinositide 3-kinase (PI3K) was discovered. After several decades of research, the PI3K/AKT pathway is of great research value due to its extensive involvement (39, 40). The PI3K/AKT signaling pathway acts as an important part in key cell processes, such as growth, lipoprotein metabolism, protein synthesis, cell proliferation and apoptosis (41). Interestingly, the activation of the PI3K/AKT pathway inhibits hepatic gluconeogenesis (42) and according to the sequencing results, the differential gene enrichment in PI3K/AKT related signaling pathways, including Pik3cg, Pik3r3, Socs1 and other related genes, were significantly expressed after DCI treatment. Based on this, we put forward relevant research directions in the function mining of DCI. The possible regulatory molecular mechanism of DCI is associated with PI3K/AKT related pathway, which regulates the expression of other related nodes on the whole signaling pathway and to improve hepatic insulin resistance. Related differential genes are also abundantly enriched in the glycogen synthesis/gluconeogenesis pathway and many studies have demonstrated that the gluconeogenesis pathway plays a decisive role in hepatic insulin resistance (43).

The kidney and liver are the main sites of endogenous glucose production, but only the liver can respond to insulin by regulating glucose levels (44). In the fasting state, the energy supply of the human body is mainly achieved by the dynamic regulation of glycogen synthesis and decomposition by the liver (41). The glycogen synthesis and decomposition are transported to various tissues of the human body; while, inhibiting the synthesis of new fatty acids. After eating, the human body controls the level of hepatic glucose production (HGP) through the PI3K/AKT pathway and stores glycogen for other tissues with sufficient energy. The *G6pc* and *Pklr* genes are enriched in the gluconeogenesis pathway. The levels of *G6pc* and *Pklr* genes were significantly down-regulated after DCI intervention, indicating that DCI can reduce gluconeogenesis and increase glycogen synthesis, thus improving glucose uptake and promoting peripheral insulin resistance. In addition, studies have found that the effect of DCI on hepatic glucose output and its significant consumption in metabolic diseases (45, 46), can help in explaining the antidiabetic and insulin sensitivities of DCI (47, 48). Therefore, we hypothesize that the inhibition of gluconeogenesis may be a biological process, caused by the effects of DCI on hepatic insulin resistance. In the path rich after DCI intervention, *Pik3cg, Pik3r3* and *Socs1* have higher node scores. The PI3K/AKT signaling pathway regulates a variety of cellular and biological processes and multiple functional activities. Therefore, the regulation of this pathway is associated with the improvement of various diseases, such as obesity, diabetes and cancer. Under normal conditions, the PI3K/AKT pathway is activated when regulating body functions. The PI3K/AKT pathway is inhibited when excessive energy intake occurs. The relief of obesity and insulin resistance is through the activation of the PI3K/AKT pathway. When the PI3K regulation mechanism is disturbed, an abnormal expression occurs, that lead to the occurrence of many human diseases, such as obesity and cancer (49). Studies have found that the PI3K inhibition is effective in preventing obesity, which greatly reverses human diseases (50). In summary, the three DEGs, that we have enriched and screened, play a key regulatory role in the process of alleviating the disease, and play a vital role in understanding the biological activity of DCI. We are more convinced that DCI is in the liver. As there is a certain regulation in insulin resistance.

Studies have found that insulin inhibits the expression of PEPCK and G6PC. PI3K/AKT pathway increase the hepatic gluconeogenesis (51–53) and insulin reduces the expression of gluconeogenesis-related genes (54). Based on these results, AKT is not only an essential regulator of insulin, but may also stimulate the extrahepatic insulin to regulate hepatic glucose metabolism by other means. Three pathways may mediate the effects of cellular involuntary effects on hepatic glucose metabolism (55). First, the expression of G6PC and hepatic glucose (HGP) in the liver is reduced by the action of insulin in the K (ATP) channel. Subsequently, insulin controls this process through the PI3K-PIP3 signaling pathway in the AgR-related peptide (AgRP) neurons (56). Secondly, insulin inhibits lipolysis through the AKT pathway, reduces circulating FFA levels in adipose tissue, and inhibits HGP in the liver even in the absence of hepatic insulin signaling. Third, insulin function prevents the α-cells from controlling glucagon secretion in the brain *via* the AKT pathway, which reduces liver HGP levels (57). All three pathways require insulin to regulate HGP *via* PI3K. Our study also found similar pathways and sites of action through the biosignal analysis.

Our research has demonstrated the effect of D-chiro-inositol on the PI3K/AKT pathway-related genes and it has been reported that the PI3K/AKT pathway also mediates lipid synthesis. The steroid regulatory element binding protein (SREBP-1c) is a transcription factor that regulates cholesterol and phospholipid metabolism and plays an important role in insulin signaling. In the liver, the overexpression of the *SREBP-1c* gene is prone to lipogenesis but does not affect cholesterol synthesis. Studies have shown that AKT can affect the expression of *SREBP-1c* gene in the liver through the regulation of the mTORC1-S6K1 pathway (58). The role of FoxO1 in hepatic lipid metabolism is less studied and some studies have shown that FoxO1 is directly involved in the insulin-promoting expression of genes in the liver (59–61). However, our study found that FoxO1 can regulate HGP and provide additional results on the relationship between PI3K/AKT and FoxO1.

Many studies have shown that *PTP1B* has an important effect on the regulation of sugar homeostasis. For this reason, we have positioned this site as an important target site for affecting obesity or for the regulation of glucose homeostasis. The related regulation of *PTP1B* will affect the downstream expression of PI3K to some extent. *XBP1S* plays an important regulatory role in endoplasmic reticulum stress. Many studies reported that *XBP1S* can participate in the regulation of many cellular and biological activities, including cell differentiation, protein secretion and apoptosis. *XBP1S* plays a protective role and belongs to the positive regulators of cytokines. At present, many studies are shifting the focus of *XBP1S* research to the regulation of glucose homeostasis. *XBP1S* is closely related to the development of T1D, T2D and insulin. Reduced sensitivity, metabolic disorders and other processes can play a corresponding role. The *FABP4* gene is a lipid carrier that is involved in the regulation of the metabolic syndrome and inflammation, and which improves insulin sensitivity. It is a potential target for the treatment of type 2 diabetes. *G6Pase* is a key rate-limiting enzyme that regulates hepatic gluconeogenesis. The *G6Pase mainly controls the release of glycogen stored in the liver and the main function is to* release glucose from the liver into the blood to regulate the body’s energy. *Pepck* also has a role in the regulation of gluconeogenesis, by effectively utilizing non-sugar substances in the process of raising blood sugar and achieving its active transformation and energy supply.

In patients with metabolic diseases caused by obesity, the reduction of FFA absorption and glucose utilization by the body fat tissue leads to ectopic accumulation of lipids in other tissues. Ectopic accumulation of fat is the main cause of insulin resistance in the body. Abnormal elevation of FFAs increases DAG levels, resulting in massive expression of acetyl-CoA in the liver, which in turn activates pyruvate carboxylase, promotes gluconeogenesis, inhibits PI3K/AKT pathway signaling and aggravates insulin resistance (62). Chronic metabolic inflammation and endoplasmic reticulum stress can also impair hepatic insulin-AKT signaling pathway and subsequent insulin resistance (63, 64). This provides a powerful basis for to study the mechanism of hepatic insulin resistance. DCI can effectively improve the role of glucose in energy supply by inhibiting oxidative stress, and improve the effect of insulin. In a related clinical study conducted on women of childbearing age, it was found that women at this stage are very likely to cause Polycystic ovary syndrome (PCOS), and the main cause of PCOS is insulin resistance. Oral DCI can improve the patient’s symptom (65). Researchers such as Yoshida K pointed out that the main reason for inducing PCOS is the decreased sensitivity of insulin in regulating the body’s blood glucose homeostasis and various tissues and organs, which hinders the signal transmission process (66). Oral DCI can enhance insulin sensitivity and improve PCOS (67). When PCOS is relieved, the ovarian function will be restored to its original function. The ovaries can well control the body’s male hormones under normal physiological functions (68, 69). Secretion, which solves the urgent need of people with PCOS, and also points out the direction for the next in-depth study of DCI.

## Conclusion

Based on the DEGs obtained by transcriptome sequencing and analysis, a regulatory network was constructed and the corresponding biological functions of the relevant insulin-related signaling pathways were analyzed. The results showed that DCI played a pivotal role in the hepatocellular insulin resistance of PA through the IRS-2-PI3K/AKT pathway and that *PTP1B, XBP1S, SREBP-1c, FABP4, G6Pase, Pepck* and other genes were potential sites in dietary supplements.

## Data Availability Statement

The original contributions presented in the study are publicly available. This data can be found here: https://www.ncbi.nlm.nih.gov/bioproject/739485, accession PRJNA739485.

## Author Contributions

C-pF contributed to the conception of the study. FC, M-cC, J-lM, J-yL, and Y-fC performed the experiment. FC, C-pF, S-jY, and J-lC contributed significantly to analysis and manuscript preparation. FC performed the data analyses and wrote the manuscript. S-jY, and J-lC helped perform the analysis with constructive discussions. All authors contributed to the article and approved the submitted version.

## Funding

The research were supported by the Science and Technology Innovation Project of Shanxi Agricultural University (2020BQ82).

## Conflict of Interest

The authors declare that the research was conducted in the absence of any commercial or financial relationships that could be construed as a potential conflict of interest.

## Abbreviations

PKC, protein kinase C; InsR, insulin receptor; PI3K, phosphoinositide-3-kinase; AKT, protein kinase B; FOXO1, fork-head frame protein O1; G6Pase, glucose-6-phosphatase; PTP1B, Protein Tyrosine Phosphatase 1b; XBP1, X-box binding protein1; FABP4, fatty acid-binding protein 4; SREBP-1c, sterol regulatory element binding protein-1c; PEPCK, phosphoenolpyruvate carboxykinase; DCI, D-chiro-inositol; IPG-P, inositol phosphoglycan phosphatase; PDK, pyruvate dehydrogenase kinase; PDH, pyruvate dehydrogenase complex; IRS-2, insulin receptor substrate proteins 2; PA, palmitic acid; DAG, diacylglycerol.
